# Gram Negative Wound Infection in Hospitalised Adult Burn Patients-Systematic Review and Metanalysis-

**DOI:** 10.1371/journal.pone.0095042

**Published:** 2014-04-21

**Authors:** Ernest A. Azzopardi, Elayne Azzopardi, Liberato Camilleri, Jorge Villapalos, Dean E. Boyce, Peter Dziewulski, William A. Dickson, Iain S. Whitaker

**Affiliations:** 1 Institute of Life Science, Swansea University College of Medicine, Singleton Park, Swansea, United Kingodm; 2 The Welsh Centre for Burns and Plastic Surgery, Moriston Hospital, Swansea, United Kingdom; 3 Research Institute for Health and Social Change, Faculty of Health, Psychology and Social Care, Manchester Metropolitan University, Elizabeth Gaskell Campus, Manchester, United Kingdom; 4 Department of Statistics and Operations, Tal-Qroqq Campus, University of Malta, Msida, Malta; 5 Department of Burns and Plastic Surgery, Chelsea and Westminster Hospital NHS Trust, London, United Kingdom; 6 St. Andrews Centre for Burns and Plastic Surgery, Chelmsford, United Kingdom; University of Ulster, United Kingdom

## Abstract

**Background:**

Gram negative infection is a major determinant of morbidity and survival. Traditional teaching suggests that burn wound infections in different centres are caused by differing sets of causative organisms. This study established whether Gram-negative burn wound isolates associated to clinical wound infection differ between burn centres.

**Methods:**

Studies investigating adult hospitalised patients (2000–2010) were critically appraised and qualified to a levels of evidence hierarchy. The contribution of bacterial pathogen type, and burn centre to the variance in standardised incidence of Gram-negative burn wound infection was analysed using two-way analysis of variance.

**Primary Findings:**

*Pseudomonas aeruginosa, Klebsiella pneumoniae, Acinetobacter baumanni,* Enterobacter spp., Proteus spp. and *Escherichia coli* emerged as the commonest Gram-negative burn wound pathogens. Individual pathogens’ incidence did not differ significantly between burn centres (F (4, 20) = 1.1, p = 0.3797; r2 = 9.84).

**Interpretation:**

Gram-negative infections predominate in burn surgery. This study is the first to establish that burn wound infections do not differ significantly between burn centres. It is the first study to report the pathogens responsible for the majority of Gram-negative infections in these patients. Whilst burn wound infection is not exclusive to these bacteria, it is hoped that reporting the presence of this group of common Gram-negative “target organisms” facilitate clinical practice and target research towards a defined clinical demand.

## Introduction

### 1.1 Background and Rationale

Gram-negative infection is a global health concern [1]. Several advances have been registered in the field of intensive care, ventilatory support, skin substitution and fluid balance [2]. However, infection has emerged as a major, often unmitigated complication in burn injury, which incurs significant morbidity, mortality and healthcare cost [3]. Management of acute infection in thermal injury presents unique challenges in terms of clinical diagnosis and rapid institution of effective antimicrobial chemotherapy. Clinical diagnosis is hampered by thermal injury-induced hyperpyrexia, immune suppression, and systemic inflammatory response syndrome [3], [4]. These factors make clinical diagnosis difficult and promote infection [5]. It is, however, well-established that Gram-negative pathogens predominate beyond the early post-burn period [5]. Centres for Disease Control (USA), the British Society of Antimicrobial Chemotherapy, and its European and Asian Counterparts provide extensive documentation regarding aetiological profiles and incidences of the major protagonists in other disease, such as pneumonia and urinary tract infection [6], [7]. This data, in turn, provides aetiological targets for rationalised expedited, targeted antimicrobial prescribing, infection control, and antimicrobial development [8]. Traditional teaching is based on incidence data that is non-standardised and difficult to compare; it maintains that Gram negative burn wound isolates differ between burn centres [9]–[11].

## Objective

The purpose of this study is to establish whether the isolates associated with clinical Gram negative burn wound infection differ between burn centres. This study also sought to establish standardised incidence rates for the organisms identified.

## Methods

### 3.1 Aim Construct

The terms of reference in relation to this systematic review and meta-analysis (File S1) in Patient Intervention Comparator and Outcome (PICO) format, are reported in File S2, in conformance to pre-validated criteria [12].

### 3.2 Literature Search

A combination of National Library of Medicine (NLM) Medical Subject Heading (MeSH) Descriptor Data Browser terms were used to increase search sensitivity [13]. These were used in a first generation electronic search whose results were manually screened for relevance (File S3). The first generation search was performed using the OVID-SP and PUBMED platforms (File S4). The second generation search involved manual back-referencing and Web of Knowledge. Results of the electronic literature search are reported extensively in File S5.

### 3.3 Inclusion/Exclusion Criteria

This study specifically adhered to the PICO-specified terms of reference as inclusion criteria (File S2). In order to enable data pooling and anlalysis, only primary literature investigating adult hospitalised burn patients only was analysed, and studies including patients with delayed transfer were excluded from this analysis. Primary literature published between 2000 and 2010, in English, studying adult locally-injured hospitalised humans only was included. The limitations of this approach are fully acknowledged, and applicability of the study to other populations is debated in the discussion section.

### 3.4 Reporting of the Systematic Review and Evidence-based Process

PRISMA guidelines were applied to report the systematic review and evidence-based process. The retrieval process reported heterogeneous methodologies in the primary literature but no randomised controlled trials (RCT’s), requiring these guidelines to be adapted. Systematic review of studies other than RCTs is not new [14]. To ensure comparability and adequate data selection rigorous critical appraisal [15], [16] was applied to determine the quality of the primary studies retrieved, and ensure inclusion of comparable, current, valid and relevant evidence [17]. Pre-validated critical appraisal tools were employed on the primary research to achieve significant depth of appraisal [12], [18]. Two researchers, arbitrated by a third, independently performed critical appraisal. The Oxford Centres for Evidence Based Medicine “levels of evidence” framework was used to provide a framework to reflect the robustness of individual studies [19]. The literature retrieval process is reported in detail (Files S5, S6).

### 3.5 Operational Definitions and Summary Measures

Primary literature reported various measures of incidence, therefore incidence rates were standardised as number of new cases per 1000 patient-years [20]. As a working definition, an organism was defined as causative if it could be discerned from the study that the organism was isolated from wound in the presence of clinical infection. “New” was defined as the first documentation of an organism, thereby excluding relapse or re-infection. Clinical diagnosis of infection was based on reconciliation of primary literature to the definitions of Greenhalgh *et al.* [21]. For the purposes of this study, studies reporting on patients whose transfer to the definitive treatment centre was delayed (non-immediate) were excluded.

### 3.6 Statistical Analysis

Statistical analysis was performed with Graphpad Prism v5 for windows (CA, USA). The contribution of the two independent variables under consideration (bacteria, burn centre) to the variance in standardised incidence of Gram-negative burn wound pathogens was analysed using two-way analysis of variance (ANOVA). Statistical significance was assumed when p<0.05. Statistical analysis was performed blinded, by a qualified statistician (LC).

## Results

### 4.1 Literature Retrieval and Critical Appraisal

**Figure 1:**
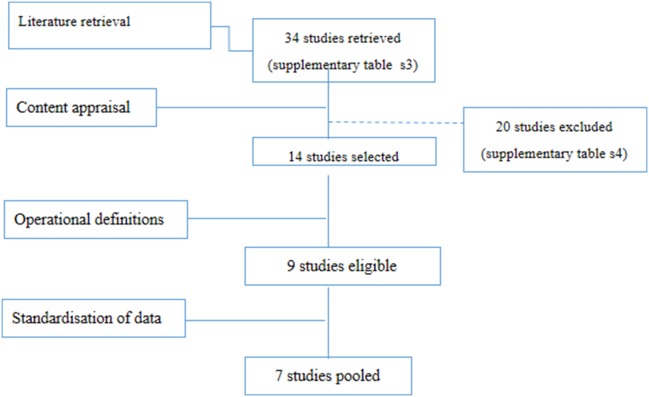
PRISMA-style scheme reporting the literature retrieval and selection strategy arriving to the final 7 studies whose data could be pooled for statistical analysis.

**Figure 2:**
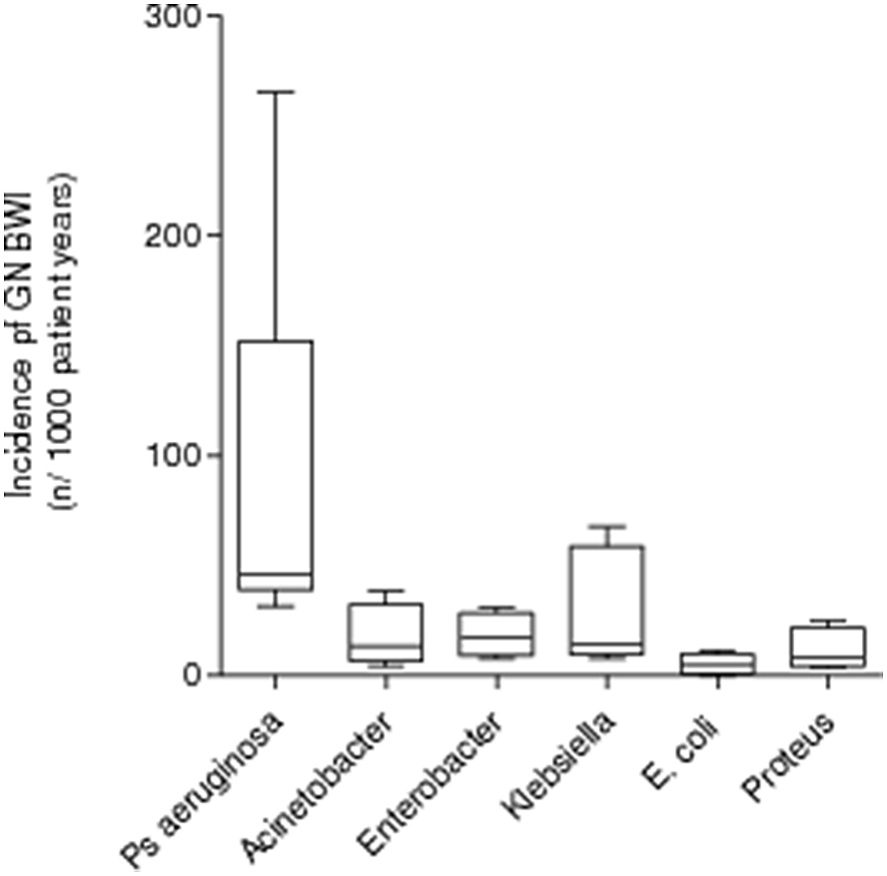
Box-whisker plot reporting data dispersion for the standardised incidence of Gram-negative burn wound injury in civilian adult hospitalised patients. Data shown represents mean±1SD.

**Figure 3:**
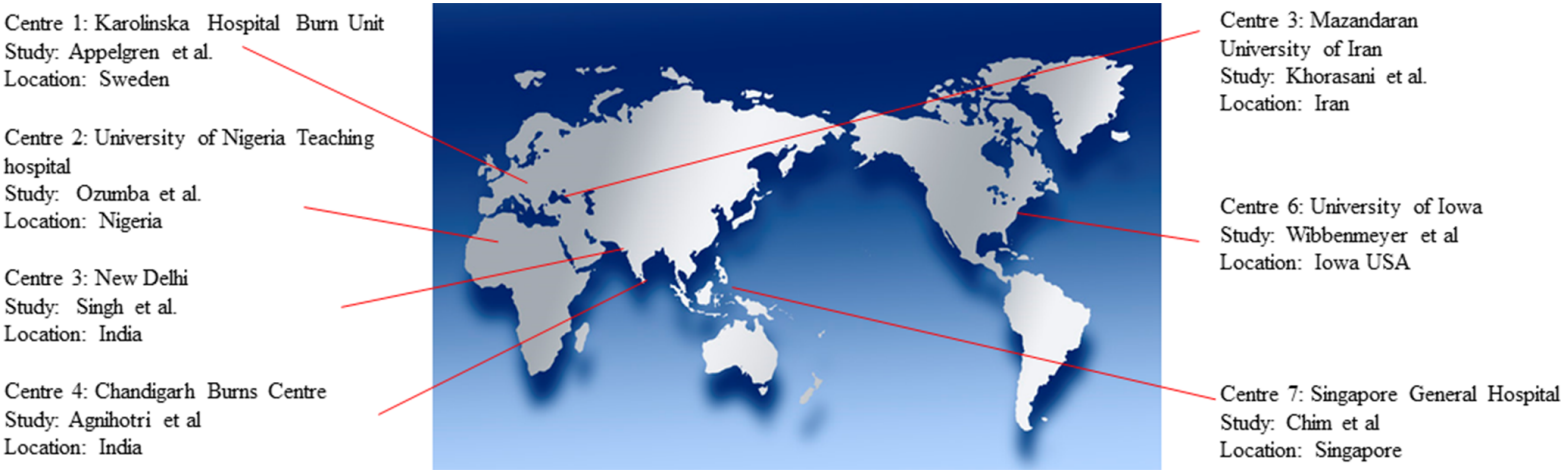
Geographic distribution of the centres from which studies were critically appraised. The study design and sample size are provided in figure 2.

Thirty four studies were retrieved by the initial literature search (Figure 1). Narrative critical appraisal is provided in extensive detail in File S7. Of these studies, 20 did not conform to the inclusion criteria and were excluded (File S6). Fourteen studies were included for critical appraisal (Table 1). A further 5 studies did not conform to operational definitions (section 3.3). “Strength of the evidence” underpinning the remaining studies was evaluated by critical appraisal and is reported in Table 2. Two further studies [22], [23] investigated primary incidence over week 1 post-burn only. Because of this methodological heterogeneity, their results could not be pooled or confidently compared to the rest of the literature. Standardised incidence data from the remaining 7 studies (Table 2) was extracted and pooled (Figure 2). The geographic location of the burn centres from which the data was pooled is reported in Figure 3.

**Table 1:** Literature Included for Critical Appraisal.

n	Reference	Design	Aim	Sample
1	[50]	Retrospective study	“To determine the bacterial profile and antimicrobial susceptibility of the isolates and todescribe the change in trends over the study period.”	665
2	[51]	Retrospective Cohort	“To determine the incidence and cause of nosocomial infections in all patients admitted toour burn intensive care unit (BICU) over a 5-year period”	76
3	[52]	Prospective	Describe a specially designed computer system for the analysis of data, and report theresults from the first 3 years of using the system for routine registration of infection in aconsecutive series of burn patients.	83
4	[9]	Retrospective	“To determine the changing patterns and emerging trends of bacterial isolates and theirantimicrobial susceptibilities”	759
5	[53]	Retrospective cohort	“To analyse the bacterial isolates from the wounds of patients admitted to the BurnsUnit and to determine the sensitivity pattern of the commonly cultured organisms’	336
6	[11]	Prospective Study	“To investigate the profile of micro-organisms and resistance to antimicrobial agentsin a tertiary referral burn centre”	113
7	[54]	Retrospective Study	“To determine the bacterial profile and antibiotic susceptibility pattern of burnisolates at the Queen Elizabeth Central Hospital (QECH), Blantyre”	317
8	[23]	Prospective	“To determine nosocomial infections in the Tohid Burn Centre in Tehran, Iran”	582
9	[22]	Prospective Clinical Audit	This prospective clinical audit investigated the primary incidence of BWI between theusual burn patients […] and a number of survivors from the Bali bombings duringa 3-month audit.	64
10	[55]	Retrospective Cohort Study	‘To document burn wound infection and problems faced by the clinicians’	71
11	[56]	Narrative review	An index case of pseudomonal BWI is reported followed by a narrative review ofincidence mortality, risks and prognosis	N/A
12	[57]	Narrative Review	A narrative review describing risk two *Acinetobacter baumanni* outbreaks, and riskfactors [aim not explicitly stated]	72
13	[33]	Case-control arm Retrospective Cohort Arm	“This study was conducted to determine the risk factors for acquisition ofimipenem-resistant *Pseudomonas aeruginosa* (IRPA) in the burn unit.”	370
14	[6]	Prospective Cohort Study	‘To determine accurate infection rates, risk factors for infection, and thepercentage of infections.’	157

**Table 2:** Incidence of Gram negative organisms causing clinical burn wound infection extracted from eligible primary literature.

Study centre	Evidence Level	Patients (n)	Study Duration (Years)	BWI Incidence	Gram-ve BWI incidence	*P. aeruginosa*	*A. baumanni*	*Enterobacter spp.*	*K. pneumoniae*	*E. coli*	*Proteus spp.*	Mixed I Infection (%)
[50]	4	692	5	192	148	111	13.8	7.5	7.5	5.0	6.2	13%
[52]	2b	230	3	155	70	39	4.3	14	11.5	0	6.0	12%
[51]	3	57	5	163	89	31.4	38.4	21	14	3.5	3.5	NR
[11]	2b	113	0.5	530	NR	265*	NR	NR	NR	NR	NR	NR
[55]	4	71	5	253	174	39.4	30	30.8	67.6	11	25.2	6.1%
[9]	4	759	5	151	86.2	46	13.4	30	50	5.6	10.4	40%*
[6]	2b	157	1	382	NR	152	NR	NR	NR	NR	NR	NR

(New Cases per 1000 patients per year). Average rate of Gram-negative BWI 156 (hospitalised adults);

Percentage of BWI in hospitalised adults due to the identified bacteriological profile = 64.1%;

NR: not reported.

*Outlying variable.

### 4.2 Data Extraction

Data from clinically and statistically comparable studies was transformed to a standardised incidence rate (n new Gram negative BWI per 1000 patient-years, Table 2). Data-distribution of the standardised incidence rates for each pathogen in the identified Gram-negative BWI profile is represented by a box-whisker plot (Figure 1). Descriptively, all these studies reported a similar set of bacteria consisting of *P. aerugionsa, K. pneumoniae, E. coli, Enterobacter spp. and Proteus spp.* as the commonest Gram-negative pathogens to be isolated from clinically infected burn wounds in the burn centres studied. Standardised incidences reported in this study report *Pseudomonas aeruginosa* is the commonest BWI pathogen, but the incidence varied widely and differs significantly from the preceding literature [24].

### 4.3 Statistical Analyses

The majority of infections, 60.2%±12.5% (mean±1 SD) were Gram-negative. A mean incidence rate of 156 new Gram-negative BWI per 1000 patient-years was calculated. Mixed infection accounted for 10.3±3.7% (mean±1 SD) of infection. Two-way ANOVA reported that identity of the bacterial species (independent variable) was responsible for 47.8% of the total variance (F (5, 20) = 4.13). This was highly statistically significant (p = 0.0098). The same statistical method also reported that Burn Centre accounted for 9.84% of the total variance (F (4, 20) = 5.11), but this effect was not statistically significant (p = 0.3797).

## Discussion

This study studied primary literature to establish whether the isolates associated with clinical Gram negative burn wound infection differ between burn centres. Burn wound infection has traditionally been a difficult area for the clinician, and one of the primary reasons is the difficulty in knowing which organisms to target. This study established that organisms causing Gram-negative burn wound infection do not differ significantly between burn centres. Analysis of the standardised data confirm, within this study’s limitations, the principal hypothesis that the organisms causing Gram-negative burn wound infection are similar, regardless of geographic location of the treating centre (p<0.05). This finding presents a significant departure from traditional teaching regarding the behaviour of infected burn wounds (section 1). The lack of standardised data reporting may be one possible explanation for this discord [25]. In contrast, aetiological profiles and incidences of the major protagonists in other disease such as urinary tract infection have long been established, and are constantly reviewed [6], [7]. Establishing the organisms that commonly cause Gram-negative infection in burn wounds may confer to acute burns patients the long-term benefits enjoyed by these other common diseases, such as rationalised, expedited, targeted antimicrobial development, prescribing, infection control, and surveillance of resistance patterns. These findings, integrated with local data regarding susceptibility patters, may facilitate the formulation of “first line” antibiotic treatment strategies, and increase probabilities of therapeutic efficacy. For example, worldwide resistance to commonly used broad spectrum antibiotics such as ceftriaxone is common (from 16.3% E. coli to 64% of Acinetobacter strains studied) [26].

Identifying this set of bacteria may also have implications on defining research strategies in drug redevelopment. Only up to 1.4% of isolates from across the species identified are resistant to colistin, emphasising the usefulness of this antibiotic as a drug of last resort [26]. These observations lend further credence to the strong interest of this venerable class’s redevelopment via semi-synthetic chemistry approaches. The approach to identifying a common set of bacteria responsible for burn wound infection has already borne translational fruit. Recently, an in-depth analysis for commonalities of biochemical and virulence mechanisms involved in the aetiology of infection with these organisms identified that substantial production of bradykinin is common to all these pathogens and leads to enhanced vascular permeability and sequestration of macromolecules. Based on this principle, we recently published evidence of a novel, size-based paradigm for drug targeting in infection [27]; statistically optimised a bioresponsive polymeric payload carrier to achieve this goal [28]; proposed a novel class of macromolecular antimicrobial agents capable of locally triggered enzymatic activation at the infected site, retaining antimicrobial potency to match the conventional clinical equivalent whilst significantly reducing *in vivo* toxicity [29], [30].

Ample evidence exists to support the notion that morbidity, mortality and quality of life outcomes in burn patients is associated to the organisms identified in this study. For example, wound infection with *P. aeruginosa*, *E. coli*, and *K. pneumoniae* wound is an independent predictor of mortality [3], [31]. These bacteria also promote failure of healing [32] which is of major consequence to the management of extensive burn wounds by serial excision where time to healing is essential and donor sites for grafting come at a premium. Moreover, specific risk factors associated to burn wound infection with these organisms have been identified [33]–[35], and modification of management practices may lower infection rates resulting in improved outcomes. The importance of identifying this set of organisms as the prime perpetrators of Gram-negative burn wound infection, regardless of the treating centre, is therefore apparent.

*P. aeruginosa* (Figure 2) exhibits an interesting, wider dispersion of dispersion of data compared to the other organisms. A possible explanation lies in the presence of an outlier data set [11] (Table 2), hence the long superior whisker for the *P. aeruginosa* plot in Figure 2. However ANOVA, is remarkably robust to moderate departures from normality caused by outlier data sets. One possible explanation for this outlier may lie in the susceptibility of the standardised incidence of *P. aeruginosa* to the overall infection rate reported from the relative burn centre. In fact, the overall infection rate reported from this centre is also high [11].

Such a study presented unique difficulties. Mere presence of organisms on a wound does not imply infection. Histological documentation of infection into viable tissue may secure the diagnosis. However, in practice, few if any centres worldwide have the substantial resources required to fulfil laboratory diagnostic criteria such as routine microbiological tissue histology and electron microscopy on a daily basis. Moreover, definitions of burn wound infection underpinned by these (tissue biopsy and electron microscopy) laboratory investigations are largely considered dated [36], [37]. As a working definition, an organism was defined as causative if it could be discerned from the primary literature that the organism/s was isolated from the wound in the presence of clinical infection. Clinical diagnosis of infection was based on reconciliation of primary literature data to the definitions of Greenhalgh *et al.* [21]. Studies including re-infection or relapse in their incidence rates were excluded. Whilst the clinical surrogates presented in these definitions may be less specific then exhaustive (but rarely performed) laboratory investigations, the approach presented herein would reduce the potential bias presented by the resources available to the researchers producing the primary literature.

As no RCTs were identified, the conduct of this review was adapted to consider the pooling of data from primary studies with heterogeneous methodologies. Systematic review of studies other than RCTs is well-established in the literature [21]. Therefore, a rigorous critical appraisal process qualified the “strength of the evidence” underpinning the primary data included in the statistical analysis. The critical appraisal process also facilitated the reconciliation of the primary literature to the operational definitions, ensuring comparability of the studies. Such an evidence-based approach is not new [12]. Quality of the evidence provided by the primary literature was limited (Table 2), justifying the use of an evidence-based methodology combined to the systematic review process. Since the burn centres in the primary literature were geographically separate and did not have overlapping catchment areas, it was reasonable to assume that “no interaction” occurred between the two independent variables studied.

The selection of adequate inclusion and exclusion criteria was challenging. A rigorous approach in data retrieval was observed and catalogued to prevent retrieval bias. In order to minimise the possibility data contamination with lurking variables, stringent criteria were applied. Evidence exists that delayed transfer may influence the bacteriological flora burned patients [38]. Studies concur that delayed presentation may alter the pathological flora on a burn wound [3], [39]. Military patients may pass through multiple facilities to the definitive site of treatment, and this delay presented a plausible lurking variable [38], [40]. This data was therefore excluded purely as a necessity to safeguard methodological rigour rather than implying a difference between military and civilian populations. In fact, data from Operation Iraqi Freedom and Operation Enduring Freedom suggests that similar bacterial flora is present in such patients, [38], [40], [41]. Indeed primary studies describing civilian injuries [22], [42] were also excluded for similar reasons.

A five to ten year cut-off for the retrieval of primary data is well-established in evidence-based methodology [12], [43]. Such time-limits for the inclusion of primary data in evidence-based medicine harks back to the main works of Archie Cochrane and DL Sackett [44], [45], affirmed in the Sicily statement for evidence-based research [17] and cited in multiple works since then [46]–[48]. Moreover until the 1980’s burn wounds were treated by the exposure method, with application of topical antimicrobials to the burn wound surface and gradual debridement with immersion hydrotherapy [24]. Thereafter, early burn wound excision and wound closure became the focal point of burn wound management, accompanied by a change from immersion hydrotherapy to showering hydrotherapy, and a consequent decrease in the rate of burn wound infection [24]. It is also well-established that early excision has reduced the incidence of invasive infection, as underscored by key publications immediately preceding our cut-off point for retrieval of primary literature. Pruitt et al. in particular, assert that the change to early excision and grafting significantly changed, both qualitatively and quantitatively, the incidence and identity of organisms causing these infections and their timing [38], [49].

It was assumed that by the year 2000, this method of treatment would have been well-established, coinciding with the cut-off point determined by the evidence-based methodology. However, it is acknowledged that such arbitrary cut-off points may have led to retrieval bias in selection of primary literature.

## Conclusion

This study is the first to report that organisms causing clinical Gram-negative burn wound infection do not differ significantly between burn centres. Therefore, these findings establish a “target set” of Gram-negative pathogens for antimicrobial development and timely, effective treatment. *P. aerugionsa, K. pneumoniae, E. coli, Enterobacter spp. and Proteus spp.* were identified as the commonest Gram-negative pathogens to be isolated from clinically infected burn wounds regardless of the treating centre. It is of course acknowledged that other bacteria may infect the burn wound and it is especially important to monitor emerging infections [51]. However, we hope that finding a “target” set of pathogens may contribute to timely clinical treatment, effective, and clinically oriented antibiotic development. The threat posed by multi-drug resistant pathogens continues to increase, however research and clinical management in this specialist field remain poorly funded, fragmented and oftentimes reported in isolation. The paucity of relevant literature highlighted in this study illustrates the dire necessity for further epidemiological multi-disciplinary collaboration. We augur that the identification of a common aetiological Gram-negative burn wound profile will be a first step in this direction.

## Supporting Information

File S1 **PRISMA Checklist.**
(DOC)

File S2 PICO* framework applied to the study question.
(DOCX)

File S3 **Search Terms Used in the Literature Search.**
(DOCX)

File S4 **Databases Searched Electronically.**
(DOCX)

File S5 **Results of the Electronic Literature Search.**
(DOCX)

File S6 **Studies excluded after content analysis.**
(DOCX)

File S7 **Detailed critical appraisal of the literature.**
(DOCX)
