# Supplementary material for: Gram Negative Wound Infection in Hospitalised Adult Burn Patients-Systematic Review and Metanalysis-
Source: PLoS One. 2014 Apr 21;9(4):e95042. doi: 10.1371/journal.pone.0095042 (PMC3994014; doi:10.1371/journal.pone.0095042)
Supplement: File S2 — PICO* framework applied to the study question. (DOCX) [file pone.0095042.s002.docx]

| Table S1: PICO* framework applied to the study question | |
| --- | --- |
| Frame/ Mapped Semantic Class | Intention |
| Problem and Population of interest  Age; gender; treatment status; disease; symptom | Hospitalised burn wound patients. Adult civilian patients. Aetiology of Gram Negative Burn Wound Infection in this population |
| Aetiology [intervention] of interest  Aetiological Agent of Interest | Gram-negative infection. |
| Comparator of Interest | Current clinical practice. |
| Outcome (intended)  Patient Outcome | Recommendations for clinical practice. |
| *PICO: patient intervention (aetiology) comparator outcome algorithm | |
