# Supplementary material for: Gram Negative Wound Infection in Hospitalised Adult Burn Patients-Systematic Review and Metanalysis-
Source: PLoS One. 2014 Apr 21;9(4):e95042. doi: 10.1371/journal.pone.0095042 (PMC3994014; doi:10.1371/journal.pone.0095042)
Supplement: File S3 — Search Terms Used in the Literature Search. (DOCX) [file pone.0095042.s003.docx]

| Table S2: Search Terms Used in the Literature Search | | |
| --- | --- | --- |
| MeSH^®^ | Free text | Limitations |
| Infect$  Gram-Negative  Hospital-Acquired  Microbiol$  Bacter$  Multi-drug AND resistance  Thermal  Wound  Injur$  Traum$  Incidence  Risk | Infect* AND disorder  Gram AND negative  drug AND resistance AND multiple [MDR]  Burn$ | 2000-2010  Articles in English  Human  Adult  Civilian  Hospitalised |
