# Supplementary material for: Gram Negative Wound Infection in Hospitalised Adult Burn Patients-Systematic Review and Metanalysis-
Source: PLoS One. 2014 Apr 21;9(4):e95042. doi: 10.1371/journal.pone.0095042 (PMC3994014; doi:10.1371/journal.pone.0095042)
Supplement: File S4 — Databases Searched Electronically. (DOCX) [file pone.0095042.s004.docx]

| Table S3: Databases Searched Electronically | |
| --- | --- |
| Platform | databases |
| Gateway: OVID SP |  |
| Evidence Based Medicine Reviews | ACP journal club; Database of Abstracts of Reviews of Effects; Cochrane Central register of Controlled Trials; Health Technology Assessments; Cochrane Database of Systematic Reviews; National Health Service Economic Evaluation; Cochrane Methodology Register. |
| Healthcare Information Service of the British Library | Allied & Contemporary Medicine Database Guide (AMED) |
| British Nursing Index | BRNI Segment  BNIB segment |
| Exerpta Medica Database (EMBASE) | EMBASE database  EMBASE drugs & Pharmacology (EMDP)  EMBASE psychiatry reports (EMPS) |
| National Library of Medicine (NLM) | Index Medicus ®  International Nursing Index ®  Index to dental literature® |
| Pre-Medline | The Ovid Medline ® In process & Other Non-indexed Citations Database consists of in –process and PubMed –NOT-Medline records from the NLM |
| International Pharmaceuticals Abstract Database | International Pharmaceuticals Abstracts database |
