# Supplementary material for: Gram Negative Wound Infection in Hospitalised Adult Burn Patients-Systematic Review and Metanalysis-
Source: PLoS One. 2014 Apr 21;9(4):e95042. doi: 10.1371/journal.pone.0095042 (PMC3994014; doi:10.1371/journal.pone.0095042)
Supplement: File S5 — Results of the Electronic Literature Search. (DOCX) [file pone.0095042.s005.docx]

| Table S4: Results of the Electronic Literature Search | | |
| --- | --- | --- |
| no. | Searches | Results |
| 1 | ((Gram and negative) or Microbiol$ or bacter$ or biol$).mp. [mp=ti, ab, sh, hw, tn, ot, dm, mf, tx, kw, ct, nm, rw] | 1874672 |
| 2 | limit 1 to human | 987867 |
| 3 | limit 2 to yr="1999 –Current | 827841 |
| 4 | infect$ or (infectious and disorder) or (hospital And acquired) or HAI .mp. [mp=ti, ab, sh, hw, tn, ot, dm, mf, tx, kw, ct, nm, rw] | 1384510 |
| 5 | (burn$ or thermal).mp. [mp=ti, ab, sh, hw, tn, ot, dm, mf, tx, kw, ct, nm, rw] | 159964 |
| 6 | 4 AND 3 AND 5 | 2843 |
| 7 | Remove duplicates from 6 | 2234 |
| 8 | 7 not CARDI$.mp. not RESP$.mp. not PNEUMON$.mp. not GASTRO$.mp. not ABDOM$.mp. not HEPATO$.mp. not NEURO$.mp. not UROL$.mp. not BADDER.mp. not URO$.mp. not GENIT$.mp. not VAGIN$.mp. not RENAL.mp. not EYE$.mp. not KERATO$.mp. not OTO$.mp. not RHINO$.mp. not LARYNGO$.mp. not ORTHO$.mp. not MUSCUL$.mp. not VENT$.mp. not UVEO$.mp. not PAROT$.mp. [mp=ti, ot, ab, sh, hw, kw, tn, | 87 0 |
| 9 | 8 not VIROL$.mp. not VIRAL.mp. not RICKETTISA$.mp. not CANDIDA$.mp. not ZOO$.mp. not CYAHAGA.mp. not FUNG$.mp. not PORCIN$.mp. not BOVIN$.mp. not Q.mp. not BURNETTI.mp. not INTRAMEDULLA$.mp. not SPINAL.mp. not ARTHRO$.mp. not TONGUE$.mp. not NORO$.mp. not GUT.mp. not MYCO$.mp. not DENGUE.mp. [mp=ti, ot, ab, sh, hw, kw, tn, dm, mf, tx, ct, nm, rw] | 600 |
| 10 | 9 not DRESS$.mp. not MICROARTICULATE.mp. not CUYAHOGA.mp. not STOMA$.mp. not ACNE.mp. not ENDOCARD$.mp. not ACTICOAT$.mp. not XENODERM$.mp. not PHOTO$.mp. not LARVA$.mp. [mp=ti, ot, ab, sh, hw, kw, tn, dm, mf, tx, ct, nm, rw] | 470 |
| 11 | 10 not HAEMO$.mp. not RICKETTSI$.mp. not VIRUS.mp. [mp=ti, ot, ab, sh, hw, kw, tn, dm, mf, tx, ct, nm, rw] | 430 |
| 12 | limit 11 to "adult (19 to 44 years)" [Limit not valid in EMBASE,AMED,British Nursing Index,CDSR,ACP Journal Club,DARE,CCTR,CLCMR,IPAB; records were retained] | 292 |
| 13 | 12 not MRSA.ti. not METHICILLIN.ti. not GRAM-POSITIVE.ti | 274 |
| 14 | 13 not HEMODIAL$.mp. not MILK.mp. not VACUOLE.mp. not FUEL.mp. [mp=ti, ot, ab, sh, hw, kw, tn, dm, mf, tx, ct, nm, rw] | 267 |
| 15 | 14 Keep: 8; 16; 17; 19; 20; 35; 44; 51; 53; 70; 77; 84; 87; 107; 118; 120; 123; 133; 138; 164; 166; 168; 190. | 34 |
