# Supplementary material for: Gram Negative Wound Infection in Hospitalised Adult Burn Patients-Systematic Review and Metanalysis-
Source: PLoS One. 2014 Apr 21;9(4):e95042. doi: 10.1371/journal.pone.0095042 (PMC3994014; doi:10.1371/journal.pone.0095042)
Supplement: File S6 — Studies excluded after content analysis. (DOCX) [file pone.0095042.s006.docx]

| Supplementary material S5: Studies excluded after content analysis. | | |
| --- | --- | --- |
|  | Study | Reason for exclusion |
| 1 | Das &Kim [58] | Does not inform the aims, outwith TOR |
| 2 | Santucci et al. [59] | Does not inform the aims, outwith TOR |
| 3 | Geyik et al. [60] | Does not inform the aims, outwith TOR |
| 4 | Rodgers et al. [61] | Does not inform the aims, outwith TOR |
| 5 | Ferreira et al. [62] | Does not inform the aims, outwith TOR |
| 6 | Song et al. [63] | Does not inform the aims, outwith TOR |
| 7 | Ekrami & Kalantar [64] | Study included data from BWI and other sources of infection such that the results could not inform the terms of reference |
| 8 | Falk et al. [65] | The study population was due to diarrhoea in a burn unit not BWI. Therefore the results could not inform the aim |
| 9 | Albrecht et al. [66] | The paper was used to inform the critical appraisal, but its aims did not inform the terms of reference as it only sought to determine the risk of mortality of Acinetobacter infection |
| 10 | Japoni et al. [67] | Not relevant to the terms of reference |
| 11 | Nasser et al. [68] | The authors investigate colonisation not BWI |
| 12 | Yildrim et al [69] | Includes paediatric and neonatal individuals in the sample |
| 13 | [70] | Sample population is not exclusively limited to adults and the results for adults cannot be separately interpreted. |
| 14 | Guggenheim et al. [71] | The study centres purely on colonisation not infection hence does not inform the terms of reference |
| 15 | Yousefi-Mashouf & Hashemi [72] | Data includes children as the commonest age group. |
| 16 | Estahbanati et al. [73] | Study sample does not specifically address an adult population |
| 17 | Chalise et al. [74] | Study includes children and adults, aetiology or incidences nor risk factors are described despite title |
| 18 | Miranda et al. [75] | Study included delayed transfer patients |
| 19 | Falk et al. [65] | Study does not differentiate between infection and colonisation or contamination |
| 20 | Erol et al. [76] | Study specifically addressed colonisation |
