# Supplementary material for: Gram Negative Wound Infection in Hospitalised Adult Burn Patients-Systematic Review and Metanalysis-
Source: PLoS One. 2014 Apr 21;9(4):e95042. doi: 10.1371/journal.pone.0095042 (PMC3994014; doi:10.1371/journal.pone.0095042)
Supplement: File S7 — Detailed critical appraisal of the literature. (DOCX) [file pone.0095042.s007.docx]

Supplementary data S6: Detailed critical appraisal of the literature

S.1.2.1 Narrative reviews

A critical appraisal of four prior narrative reviews generally informed this study about the position of other scholars in the field [10,35,77,78] enabling an extensive assessment of earlier efforts. A common concern is the lack of standardised incidence reporting, and literature retrieval which may have introduced a considerable amount of publication and selection bias. All but one [10] lacked critique of individual studies, and all failed to identify trends in infection patterns between different international centres.

All the narrative reviews generally agreed that *Pseudomonas* infection was the commonest Gram-negative BWI in hospitalised adult civilian patients. Only two studies [10,78] reported incidence data. All reviews agreed that incidence of infection changed since the introduction of early burn excision and grafting and showering rather than immersion therapy, providing further credence to the time-limits for our study’s retrieval criteria (Section 3.2). Whilst Church *et al* [77] did not report incidence rates to substantiate their findings; their methodology was more robust, featuring broad-based structured aims, and inclusion/exclusion criteria for primary studies. Furthermore, Polavarapu et al. [79] afforded inappropriate weighting to minor studies. An element of retrieval and selection bias therefore threatened this study's rigour and validity [35].

Mayhall's comparative approach [78] reported an incidence rate of 19.3 % for *Pseudomonas aeruginosa.* This review [78] reflected a greater degree of depth, synthesis and analysis of the literature than preceding reviews, including gray literature synthesized on thematically arranged concepts. However the study population was not clearly identified [15]. A reproducible literature retrieval process or critique of the methodological designs was not reported. Edwards-Jones and Greenwood’s [10] study identified new developments within burn wound microbiology. Within this review, *Pseudomonas aeruginosa* was identified as being the single most important Gram-negative pathogen to cause BWI in hospitalised adults, with an incidence rate of 20%.

S.1.2.3 Cohort Studies

Cohort studies formed the majority of the identified literature addressing the bacteriological profile of Gram-negative BWI in hospitalised civilian adults. Cohort studies are a suitable approach to investigate causality [80]. A summary of these studies is provided in Tables 7 and 8.

S.1.2.3.1 Prospective cohort studies

Prospective cohort studies are illustrated in table S5. To investigate the ‘profile of micro-organisms [...] in a tertiary burns referral centre’ Khorasani *et al.* [11] enrolled 113 adult hospitalised patients over seven months. *Pseudomonas aeruginosa* was reported as the commonest Gram-negative BWI (incidence of 14.5%). Surprisingly this was the only study not to report *Acinetobacter*. Institutional admission criteria and terms of reference were clearly defined.

In-depth description of inclusion criteria and baseline demographic characteristics; prospective cohort methodology; stepwise description of the microbiological technique and through triangulation of data from swabs and tissue cultures reflected reduced risk of confounding, risk of retrieval bias and attention to methodological rigour. Triangulation of results increased methodological rigour.

Several authors agree that quantitative modifications of surface wound swabs strongly correlate to infection [10,78,81]. Khorasani *et al.* [11] only reported incidence rates for *Pseudomonas aeruginosa*, *Acinetobacter* spp. and *Citrobacter* spp. infection. Attempts at correspondence remained unanswered at the time our study was submitted. The qualitative data reported by Khorasani *et al.* [11]. It would have been interesting to observe the effect which one Citrobacter outbreak would have caused on the overall incidence of other Gram negative pathogens during the time-period under investigation. Methodological appraisal of Khorasani *et al.* [11] is in keeping with a rigorously performed prospective cohort study (2b) [19]. Khorasani *et al.* [11] reported an apparently significant lower *Acinetobacter spp.* and increased *Citrobacter spp.* incidence. Furthermore, a contemporary quality-assessment of microbiology laboratories across Iran and reported that *Citrobacter freundii* was correctly identified in only 79.8% of cases [82], but the correct identification rate for *Acinetobacter* spp. was significantly lower at only 29.8%. The low accuracy of identification for *Acinetobacter* spp. in local labs may have resulted in methodological bias. However, as specific data for the laboratory involved was not available this possible source of bias cannot be securely ascertained.

Khorasani *et al.* [11] conducted their six month study over the winter period, which may explain their unusually low *Acinetobacter* spp. incidence rates. Several authors in the same geographical region report a significantly decreased incidence of *Acinetobacter* infection in the winter months [83-85]. Seasonal shift patterns may therefore account for this low incidence rate. Thus although individual patient follow-up was adequate, a longer study duration may have contributed to decrease the impact of this potential lurking variable and increase the study’s rigour.

Lari and Alaghebandan's [23] prospective cohort study adopted a different methodological approach. Every admitted burn patient was swabbed immediately (post-admission, and days 3, 5 and 7) according to an operational definition of BWI based on local practice during the time of the study, which could be reconciled to Greenhalgh et al.’s [86] criteria. A step-wise microbiological approach was reported from swabbing to final diagnosis, increasing reproducibility. *Pseudomonas aeruginosa*, *Acinetobacter* spp. and *Enterobacteraceae* spp. were reported as the commonest Gram-negative infections (Table 9). An extensive description of the admission criteria and the demographic characteristics of the included patients increased applicability, reproducibility and validity [87]. The authors reported a 100% catchment for the consecutive sample followed during the study period, and the drop-out rate due to death was documented. This may mitigate for the reported lack of randomisation. Selection bias would still be a possibility since the study was not blinded. The stepwise methodological approach in Lari and Alaghebandan’s [23] study design and reporting validated and referenced microbiological techniques increased reproducibility and reflected a rigorous design. The interpretation of wound swab results included clinical observations, increasing the concordance to Greenhalgh *et al.*’s [86] criteria. The authors also reported the time-frame from burn to admission: determining the interval from burn to swab-sampling reduced the confounding influence of time, reported by several authors [10,77]. However, patients referred from other burn centres, representing delayed admission, could still present a source of confounding. It appears, therefore, that this study is in keeping with level-3b evidence [19]. However, two problems were identified. First, the authors [23] investigated primary Gram-negative BWI incidence over the first week post admission (other studies followed patients from admission to discharge or death). This reduced the risk of data duplication that could have skewed other studies’ results, but it also meant that direct comparison to other studies could not be confidently performed. Secondly, this was also the only study to report blanket antibiotic prophylaxis administration. For these reasons, results could not be pooled in Figure x (box whisker plot). Attempts to contact the authors remained unanswered at the time of manuscript submission.

Appelgren *et al.* [52] reported on the incidence and possible risk factors of BWI in adult burnt hospitalised patients at the authors’ institution. To achieve this, 233 consecutive patients were followed prospectively from admission to discharge or death over a three year period. With the exception of *Escherichia coli* and *Proteus* spp. Appelgren *et al.*’s [52] results demonstrate qualitative concordance to the rest of the studies illustrated in Table x. Gram-negative BWI infection rate of 31% and a 37.5% mixed infection rate could also be derived from this study.

Appelgren *et al.*’s [52] operational definitions and contemporaneous institutional policy were concordant to Greenhalgh *et al.*’s [86] criteria for BWI diagnosis, indicating comparability. Validated microbiological identification techniques were cited increasing reproducibility. Chi-squared statistics were appropriate to the categorical data considered and Yates’ correction was used to prevent overestimation of significance, although this approach risks being over conservative [88]. Clearly, this study's [52] design indicates a robust design, compatible with a well-conducted prospective cohort study (Level 2b) [19]. Excellent quantitative and qualitative follow up; correlation between swab results and actual significance of infection was achieved by regular combined infection control/ burn surgery ward-rounds while any diagnostic doubt was clarified by multidisciplinary consensus, reducing the reporting bias that may occur when burn surgeons and infection control staff operate independently [89]. The sample attrition rate and follow-up were reported, until discharge or death. One possible limitation of the study was lack of blinding, however, it could be argued that the possibility of retrieval bias was mitigated by the automated data entry and strict protocol described.

Appelgren *et al.*’s [52] results inform this study in several ways. In concordance to the literature, BWI with *Pseudomonas aeruginosa*, *Acinetobacter* spp. *Klebsiella* spp. and *Enterobacter* spp. were reported as the commonest Gram negative BWI. Interestingly however, no *Escherichia coli* infection was reported. *Escherichia coli* are strongly associated to faecal contamination, and it may be argued that the absence of such infection is the result of the stringent infection control protocols in place at the study's Institution [90]. This was also the only European study investigating the long term infection incidence in a burn unit, emphasizing the need for further research in this area.

A further prospective cohort study by Chim *et al.* [42] studied 57 adult civilians admitted to a burn unit over 5 years to study the incidence and explore the cause infections in burn patients. As in previous studies [23,52], background characteristics of the cohort studied were extensively reported and compared to non-infected patients (control) admitted during the same period. Microbiological analysis was performed with validated and referenced methods, increasing reproducibility, while re-swabbing at five-day intervals until death or discharge reflected adequate follow-up. Chim *et al.* [42] reported that they applied the ‘standard CDC criteria’ of Garner *et al.*[91]. However their study design actually revealed that any patients with clinical signs of infection beyond 48 hours were included as nosocomial BWI. These were related to burn wound swabs that were being taken every 5 days in keeping with Greenhalgh *et al.*’s [86] definitions. Student’ T-test with (p<0.5) was used to compare infected to non-infected patients, reducing inter-group confounding risk, while benefitting internal validity. This test is robust to small departures from normality [92] rendering it more suitable for a small sample size of 57. The main findings of this paper, (Table 10) appear to be backed by a robust methodology in keeping with level 3 evidence [19]. Although the same Gram-negative organisms are reported for BWI as the other literature appraised, the incidence of each infection would appear to be different. Particularly, this is the only prospective cohort study which reported *Acinetobacter* as being the commonest Gram-negative BWI, and the commonest BWI overall. *Acinetobacter* is endemic to tropical climates typical of Hong Kong where this study was performed. This would be concordant to the high incidence of *Acinetobacter* spp. BWI in some studies from tropical climes, such as Perth, Australia [22]. Another possible explanation for these results could be that during their period of study Chim *et al.* [42] treated patients from the Jakarta-Marriot bombing disaster of 5 August 2003. A similar aetiological pattern was reported by Silla *et al.* [22]. The latter admitted patients from the Bali Bombings of 12 October 2002 during the course of their own investigation and reported *Acinetobacter* as the commonest BWI.

S.1.2.3.2 Retrospective Cohort Studies

Two studies investigated BWI at their institution through a retrospective cohort technique [50,53]. Although one (55) focused specifically on aerobic isolates, it was noted that both studies were performed at the same institution over an overlapping time-frame. Therefore data duplication was a possibility. In-depth appraisal of these two studies revealed strikingly similar results, but a noticeable difference in the methodological rigour and validity. Both studies retrospectively retrieved wound surface swabs during the study periods and agreed that *Pseudomonas aeruginosa* was the commonest Gram-negative pathogen, that the majority of infections were monomicrobial. Both studies reported a similar incidence of *Pseudomonas* spp. *Klebsiella* spp. and *Escherichia coli,* but Kaushik *et al.* [53] pooled all other data into an “other organisms” category, accounting for 12.5% of infections, strikingly similar to the sum total of *Acinetobacter* spp. and *Enterobacter* spp. reported by Agnihotri *et al.* [50]. It is plausible then that the difference in total is accounted for by the anaerobes excluded in one study [50]. These factors point to concordance of the true data despite the apparent differences (Table 10).

The two studies however, varied in their methodological rigour. Only one study reported follow-up by repeat wound surface swabbing until death or discharge; reported validated standardised microbial culture methods and distinguished between recurrent/relapsing infections [50]. The lack of data regarding microbiological identification, and data-handling including de-duplication exposes Kaushik *et al.*’s [53] study to the threats of methodological, observer and reporting bias, although it remained valuable to support and clarify the latter’s results given the identical institution and overlapping study period.

Ozumba *et al.* [55] investigated 71 patients over 5 years while Komolafe *et al.* [54] reported on 317 patients over 6 years. Patients were followed forward in time from a historical point (admission), a methodology consistent with a retrospective cohort approach. The long period over which these two studies took place (5-6 years) eliminated the possibility of seasonal variation [42]. Ozumba *et al.* [55] clearly defined the post-burn interval at which the burns were swabbed, reducing the confounding effect of time on the aetiological profile of BWI reported by several studies [10,77,78]. Ozumba *et al.* [55] also cited validated microbiological identification techniques that were used, increasing methodological reproducibility and rigour, in contrast to Komolafe *et al.* [54]. Due to the threats to rigour and validity arising from the risk of sampling, observer and ascertainment bias, and the risk of confounding factors, it would be difficult to include quantitative data from these studies as evidence-bases for recommendations. The qualitative Gram-negative profiles reported by both papers were in keeping to the rest of the literature.

An opportunistic sample was described. Inclusion in the study was based on availability of data rather than randomised sampling possibly introducing ascertainment bias [79]. Ozumba *et al.* [55] and Komolafe *et al.'s* [54] studies were performed in a geopolitical situation of limited technical expertise, and under-funding. It would be tempting to disregard any evidence presented from these level 4 studies [93]. However, these were the only two studies addressing Gram-negative BWI in such geo-political circumstances, justifying their inclusion in this study as 'current best' evidence.

Wibbenmeyer *et al.* [89] compared nosocomial infection rates diagnosed by burn physicians to infection control practitioners. A cohort sample of 157 patients was followed over one year and compared to controls for evidence of BWI. Rates of agreement were compared between diagnostic methods used by burn surgeons and infection control personnel. *Pseudomonas* spp. infection was also reported as the commonest BWI at the study institution. Several features indicate a rigorous methodology including explicit definition of the study sample (157 consecutive patients admitted to the study institution); the study period (one year); inclusion criteria for the study cohort and burn unit treatment policy (early excision and grafting and shower therapy), increasing reproducibility. Clinical diagnosis was augmented by correlation to surface wound swabbing, both included in Greenhalgh *et al.*’s [86] diagnostic requirements. Training of data collectors decreased the risk of inter-observer variability, increasing reproducibility and methodological rigour. Strict adherence to the operational definitions was reported. Furthermore, background characteristics between sample and control groups were thoroughly reported and compared diminishing the risk of confounding. These reported factors reflected a rigorous study and increased internal validity. Wibbenmeyer *et al.* [89] argued that common BWI definitions would decrease misreporting of BWI incidence rates, and could also decrease antibiotic over prescribing. These recommendations were backed by a robust prospective cohort methodology compatible with level 2b evidence [19].

S.1.2.4 Case-Control Studies

Singh *et al.* [9] adopted a case-control approach to investigate emerging trends of bacterial isolates by comparing BWI isolates in the same institution over two five-year periods. They reported a significant difference in infections caused by *Acinetobacter* spp., *Proteus* spp. and *E. coli* species although no major shift in the aetiological profile was reported. Matching of the control group, and sample processing in the same diagnostic laboratory reducing observer bias, reducing the potential for retrieval bias which is common in case-control studies [80,94]. However, Singh *et al.*’s [9] cases and controls were not explicitly defined in terms of co-morbidity, possibly introducing confounding in terms of total burn surface patterns, invasive devices / procedures [89], and this risk of non-equivalence between case and control increases the possibility of type one error. These concerns qualify this study as level 4 evidence [19]. Despite the possible bias data did demonstrate a similar microbiological profile to other articles in the field.

S.1.2.5 Audit Reports

The literature appraised in this study reported concordance on the common Gram-negative pathogens causing BWI, although the incidences of the different Gram negative pathogens reflected some variation between different centres. One possible reason may be the different co-aetiologies presenting with the burn patient such as co-existent poly-trauma and delayed admission. The occurrence of the Bali terrorist attacks of 12 October 2002 occurred during Silla *et al.*’s [22] prospective audit, presenting an opportunity to contrast this cohort to ‘routine’ burn wound admissions. Silla *et al.* [22] performed a comparative prospective audit of 59 patients where local civilian patients were compared to 22 patients from the Bali disaster for initial infective episodes. Admission criteria for the institution, exhaustive infection control practices, background and demographic features, a trial pilot study to reduce the possibility of intra/interobserver variation and validated their microbiological identification technique increasing reproducibility, and applicability of the results. Because Silla *et al.*[22] limited their audit to the initial infective episode data could not be pooled to the other studies but the comparative nature of Silla *et al.*’s [22] study identified significant differences between burn patients with coexistent poly-trauma and delayed referral. Chi-squared statistics were used to analyse categorical variable distribution between samples, and given the small sample sizes Yates’ test was appropriately used to reduce type two error.

A significant difference in the incidence of primary infection between ‘routine’ admissions and Bali Blast victims, including infection with *Acinetobacter* spp. was reported [22]. The scenario presented is analogous to data from the Jakarta Marriot Hotel blast incident [42]. However, Chim *et al.* [42] did not differentiate this data from ‘routine’ admissions. This observation could explain why Chim *et al.* [42] who reported *Acinetobacter* spp. as the commonest Gram-negative BWI. Silla *et al.* [22] proposed their study as a comparative prospective audit, but given the follow-up of a well-defined and matched cohort of patients forward in time it parts of their study design reflect a close similarity to a rigorously conducted prospective matched cohort study. The evidence presented would therefore be in keeping with level 2b evidence [19].

References

58. Das A, Kim KS (2000) Infections in burn injury. Pediatr Infect Dis J 19: 737-738.

59. Santucci SG, Gobara S, Santos CR, Fontana C, Levin AS (2003) Infections in a burn intensive care unit: Experience of seven years. Journal of Hospital Infection: 53(51)(pp 56-13), 2003.

60. Geyik MF, Aldemir M, Hosoglu S, Tacyildiz HI (2003) Epidemiology of burn unit infections in children. Am J Infect Control 31: 342-346.

61. Rodgers GL, Mortensen J, Fisher MC, Lo A, Cresswell A, et al. (2000) Predictors of infectious complications after burn injuries in children. Pediatr Infect Dis J 19: 990-995.

62. Ferreira AC, Gobara S, Costa SE, Sauaia N, Mamizuka EM, et al. (2004) Emergence of resistance in Pseudomonas aeruginosa and Acinetobacter species after the use of antimicrobials for burned patients. Infect Control Hosp Epidemiol 25: 868-872.

63. Song W, Lee KM, Kang HJ, Shin DH, Kim DK (2001) Microbiologic aspects of predominant bacteria isolated from the burn patients in Korea. Burns 27: 136-139.

64. Ekrami A, Kalantar E (2007) Bacterial infections in burn patients at a burn hospital in Iran. Indian J Med Res 126: 541-544.

65. Falk PS, Winnike J, Woodmansee C, Desai M, Mayhall CG (2000) Outbreak of vancomycin-resistant enterococci in a burn unit. Infect Control Hosp Epidemiol 21: 575-582.

66. Albrecht MC, Griffith ME, Murray CK, Chung KK, Horvath EE, et al. (2006) Impact of Acinetobacter infection on the mortality of burn patients. J Am Coll Surg 203: 546-550.

67. Japoni A, Farshad S, Alborzi A (2009) Pseudomonas aeruginosa: Burn infection, treatment and antibacterial resistance. Iranian Red Crescent Medical Journal 11: 244-253.

68. Nasser S, Mabrouk A, Maher A (2003) Colonization of burn wounds in Ain Shams University Burn Unit. Burns. 29 23.

69. Yildirim S, Nursal TZ, Tarim A, Torer N, Noyan T, et al. (2005) Bacteriological profile and antibiotic resistance: Comparison of findings in a burn intensive care unit, other intensive care units, and the hospital services unit of a single center. Journal of Burn Care and Rehabilitation: 26(26)(pp 488-492), 2005.

70. Altoparlak U, Erol S, Akcay MN, Celebi F, Kadanali A (2004) The time-related changes of antimicrobial resistance patterns and predominant bacterial profiles of burn wounds and body flora of burned patients. Burns 30: 660-664.

71. Guggenheim M, Zbinden R, Handschin AE, Gohritz A, Altintas MA, et al. (2009) Changes in bacterial isolates from burn wounds and their antibiograms: A 20-year study (1986-2005). Burns 35: 553-560.

72. Yousefi-Mashouf R, Hashemi S (2006) The epidemiology of burn wound infections in patients hospitalized in burn center of Hamedan, Western Iran. Journal of Medical Sciences 6: 426-431.

73. Estahbanati HK, Kashani PP, Ghanaatpisheh F (2002) Frequency of Pseudomonas aeruginosa serotypes in burn wound infections and their resistance to antibiotics. Burns 28: 340-348.

74. Chalise PR, Shrestha S, Sherpa K, Nepal U, Bhattachan CL, et al. (2008) Epidemiological and bacteriological profile of burn patients at Nepal Medical College Teaching Hospital. Nepal Med Coll J 10: 233-237.

75. Miranda BH, Ali SN, Jeffery SL, Thomas SS (2008) Two stage study of wound microorganisms affecting burns and plastic surgery inpatients. J Burn Care Res 29: 927-932.

76. Erol S, Altoparlak U, Akcay MN, Celebi F, Parlak M (2004) Changes of microbial flora and wound colonization in burned patients. Burns 30: 357-361.

77. Church D, Elsayed S, Reid O, Winston B, Lindsay R (2006) Burn wound infections. Clin Microbiol Rev 19: 403-434.

78. Mayhall CG (2003) The epidemiology of burn wound infections: then and now. Clin Infect Dis 37: 543-550.

79. Jadad A (1998) Randomised Controlled Trials. London: BMJ Publishing.

80. Crombie I (2008) A Pocket Guide to Critical Appraisal. Dundee: BMJ Publishing Group.

81. Uppal SK, Ram S, Kwatra B, Garg S, Gupta R (2007) Comparative evaluation of surface swab and quantitative full thickness wound biopsy culture in burn patients. Burns 33: 460-463.

82. Abbassi M, Rahbar M, Hekmat Yazdi S, Rashed Marandi F, Sabourian R, et al. (2006) Evaluation of the 10th External Quality Assessment Scheme results in clinical microbiology laboratories in Tehran and districts. East Mediterr Health J 12: 310-315.

83. Gales AC, Jones RN, Forward KR, Linares J, Sader HS, et al. (2001) Emerging importance of multidrug-resistant Acinetobacter species and Stenotrophomonas maltophilia as pathogens in seriously ill patients: geographic patterns, epidemiological features, and trends in the SENTRY Antimicrobial Surveillance Program (1997-1999). Clin Infect Dis 32 Suppl 2: S104-113.

84. McDonald LC, Banerjee SN, Jarvis WR (1999) Seasonal variation of Acinetobacter infections: 1987-1996. Nosocomial Infections Surveillance System. Clin Infect Dis 29: 1133-1137.

85. Perencevich EN, McGregor JC, Shardell M, Furuno JP, Harris AD, et al. (2008) Summer Peaks in the Incidences of Gram-Negative Bacterial Infection Among Hospitalized Patients. Infect Control Hosp Epidemiol 29: 1124-1131.

86. Greenhalgh DG, Saffle JR, Holmes JHt, Gamelli RL, Palmieri TL, et al. (2007) American Burn Association consensus conference to define sepsis and infection in burns. J Burn Care Res 28: 776-790.

87. Strauss S, Scott Richardson W, Glasziou P, Brian-Haiynes R, al. e (2005) Evidence Based Medicine 3rd Edition.

88. Sokal R, Rohlf F (1981) Biometry: The Principles and Practice of Statistics in Biological Research.; Freeman W, editor. Oxford: Oxford University Press.

89. Wibbenmeyer L, Danks R, Faucher L, Amelon M, Latenser B, et al. (2006) Prospective analysis of nosocomial infection rates, antibiotic use, and patterns of resistance in a burn population. J Burn Care Res 27: 152-160.

90. Greenwood D, Slack R, JF. P (2002) Medical Microbiology; Horne T, editor. Nottingham: Churchill Livingstone.

91. Garner JS, Jarvis WR, Emori TG, Horan TC, Hughes JM (1988) CDC definitions for nosocomial infections, 1988. Am J Infect Control 16: 128-140.

92. Swinscow T, Campbell M (2002) Statistics at Square One. London: BMJ Publishing Group.

93. Phillips P, Ball C, Sacket D., *al e* (2001) Oxford Centre for Evidence-based Medicine - Levels of Evidence (March 2009). In: Phillips. P, editor. Centre For Evidence Based Medicine. Oxford: University of Oxford. pp. Hierarchy of Evidence Levels.

94. Greenhalgh T (2001) How to Read a Paper- the Basics of Evidence Based Medicine. London: British Medical Journal Publishing Group.
